# Effects of an Online Community Peer-support Intervention on COVID-19 Vaccine Misinformation Among Essential Workers: Mixed-methods Analysis

**DOI:** 10.5811/westjem.2023.1.57253

**Published:** 2023-02-27

**Authors:** Dominic Arjuna Ugarte, Sean Young

**Affiliations:** University of California, Irvine, Department of Emergency Medicine, Orange, California

## Abstract

**Introduction:**

Public health efforts to reduce the spread of coronavirus disease 2019 (COVID-19) have been plagued by vaccine hesitancy and misinformation. Social media has contributed to spreading misinformation by creating online environments where people find information or opinions that reinforce their own. Combating misinformation online will be essential to prevent and manage the spread of COVID-19. It is of particular urgency to understand and address misinformation and vaccine hesitancy among essential workers, such as healthcare workers, because of their frequent interactions with and influence upon the general population. Using data from an online community pilot randomized controlled trial designed to increase requests for COVID-19 vaccine information among frontline essential workers, we explored the topics discussed on the online community related to COVID-19 and COVID-19 vaccination to better understand current misinformation and vaccine hesitancy.

**Methods:**

For the trial, 120 participants and 12 peer leaders were recruited through online advertisements to join a private, hidden Facebook group. The study consisted of an intervention and control arm, each with two groups of 30 randomized participants each. Peer leaders were only randomized into one of the intervention-arm groups. Peer leaders were tasked with engaging the participants throughout the study. Posts and comments of only participants were coded manually by the research team. Chi-squared tests assessed differences in the frequency and content of posts between intervention and control arms.

**Results:**

We found significant differences in the numbers of posts and comments focused on topics of general community, misinformation, and social support between intervention and control arms (6.88% vs 19.05% focused on misinformation, respectively, (P <0.001); 11.88% vs 1.90% focused on social support, respectively, (P <0.001); and 46.88% vs 62.86% focused on general community (P <0.001)).

**Conclusion:**

Results suggest that peer-led online community groups may help to reduce the spread of misinformation and aid public health efforts in our fight against COVID-19.

## INTRODUCTION

Misinformation has continued to plague efforts to address coronavirus disease 2019 (COVID-19)^1^,^2^ and exacerbated vaccine hesitancy due to the politicization of COVID-19.^3^–^5^ Social media has been a driver of misinformation, creating environments where people may only find information that reinforces their own.^6^–^8^ Even those in the healthcare field have been affected by this problem,^7^,^9^ but it is important that healthcare workers set an example for the public on scientifically proven options for reducing the spread of COVID-19.^10^ The Harnessing Online Peer Education (HOPE) intervention has successfully created attitude and behavior change in multiple locations and medical conditions,^11^–^13^ and this intervention may be applied to reduce misinformation and promote vaccination. The HOPE tool uses trained peer leaders to help provide support to others online.^11^–^13^

To better understand the growing problems around misinformation and vaccine hesitancy, we used data from a HOPE pilot randomized controlled trial designed to increase requests for COVID-19 vaccine information among frontline essential workers. In this study we sought to explore the topics discussed on the online community related to COVID-19 and assess differences in conversation topics and frequency between study arms.

## METHODS

### Recruitment

From July 23–August 20, 2021, participants and peer leaders were recruited online to join a Facebook group for a research study. Those who clicked on the advertisements were routed to a screening survey and, if eligible, were called by the study team to verify they were a unique person and friend us on Facebook. Participants were eligible for the study if they were ≥18 years, a US resident, an English speaker, part of phase 1a or 1b of the COVID-19 vaccine rollout (eg, healthcare workers or teachers), and someone who has not received a COVID-19 vaccine. Peer leaders matched participants in eligibility criteria and had initially been vaccine hesitant (when asked, they mentioned a reason they did not want the vaccine) but had eventually received at least the first dose of any COVID-19 vaccine (and showed us a picture of their vaccination card). Peer leaders were also required to attend three virtual training sessions (Zoom Video Communications, Inc, San Jose, CA).

### Peer Leader Training

Each session was approximately three hours. Session one focused on background information of COVID-19 and current misinformation. Session two introduced components of communication and various ways of communication online. Stigma and politicization of COVID-19 and how to address these polarizing topics were discussed. Suggested weekly topics were also introduced. We informed peer leaders that the groups were free-flowing and conversations would depend on how participants reacted and interacted with each other. Session 3 focused on the study design. Throughout training, peer leaders participated in group activities to practice using Facebook features and engaging others. After each session, peer leaders were given homework to help reinforce what they learned (eg, post a video about COVID-19 vaccine education).

### Intervention

A total of 120 participants were randomly assigned to intervention or control arms. Twelve peer leaders were randomly assigned to an intervention group. Each arm consisted of two private, hidden Facebook groups with 30 participants each. The groups in the intervention arm had six peer leaders each. The four-week study started on August 21, 2021. Twelve participants were later removed from analysis as it was discovered they had been vaccinated before the study began (six from the intervention and six from the control). Participants completed surveys at baseline and post intervention. They were told to use Facebook as they would normally and were also reminded each week that they could request information about the COVID-19 vaccine, including where to receive it. Peer leaders were responsible for reaching out to their assigned participants at least three times per week and completing a tracking sheet that documented which participants they had reached out to and whether there was any response. Each week, peer leaders also met with the study team to discuss questions or problems. Please see references for further details about HOPE studies.^11^–^13^

**Population Health Research Capsule**

What do we already know about this issue?

*Combating vaccine hesitancy and misinformation online will be essential to prevent and manage the spread of coronavirus disease 2019 (COVID-19)*.

What was the research question?

*Can peer-led online communities reduce COVID-19-related misinformation and vaccine hesitancy?*

What was the major finding of the study?

*Compared to the control group, the intervention group had less misinformation (6.9% vs 19.1%) and more socially supportive comments (11.9% vs 1.9%, both P <0.001)*.

How does this improve population health?

*The Harnessing Online Peer Education (HOPE) intervention is a promising tool to reduce vaccine hesitancy misinformation and create a supportive community environment*.

### Analysis

We manually coded posts and comments from August 21–September 17, 2021. Using a subset of 20 posts, interrater reliability for each category was calculated between the first author and another research associate in the lab to be an average Cohen’s κ = 0.59. Discrepancies were discussed and resolved and the remaining posts and comments were labeled by the first author.^11^,^14^,^15^ Post or comments could be labeled as follows; “social support” (supportive words to another member); COVID-19 (any topic about COVID-19); COVID-19 facts (scientific facts about COVID-19); COVID-19 misinformation (false or misleading information about COVID-19); COVID-19 experiences (any topic that described a participant’s or their family’s/friend’s experience around COVID-19); COVID-19 opinions (any opinion about COVID-19); COVID-19 questions (any questions about COVID-19); other misinformation (false or misleading information about a topic besides COVID-19;, misinterpreted facts (referencing an actual COVID-19 fact or research study but arriving at the wrong conclusion), and “general community” (any topic that didn’t fit in the other categories) (Table 1). For each category, respectively, Cohen’s κ = 0.64, 0.88, 0.62, 0.46, 0.38, 0.29, 0.46, 1, 0.64, 0.50. Categories were not mutually exclusive. Data were extracted and analyzed by the first author. Only posts or comments made by participants (not peer leaders) were coded and included in the analysis. We used Poisson distribution to assess differences in counts of posts and comments between arms. Chi-squared tests assessed differences in types of posts and comments. All analyses were conducted in Microsoft Excel version 1808 (Microsoft Corporation, Redmond, WA).

### Ethics Statement

This study was exempted by the University of California, Irvine Institutional Review Board.

### Results

The focus of this analysis was the online conversations. For data about the full intervention, please see our paper about the full study.^12^ During the study, there were more posts and comments in the control arm (315 vs 160 in intervention; *P* <0.001) (Table 2). Most posts and comments were about COVID-19 in both the intervention and control arms (50.63% and 54.29%, respectively) (Table 2). We found significant differences in the amounts of general community, misinformation, and social support between arms. Misinformation was 6.88% of participant posts and comments in the intervention and 19.05% of participant posts and comments in the control (*P* <0.001) (Table 2). Social support was 11.88% of participant posts and comments in the intervention arm and 1.90% of participant posts and comments in the control arm of the study (*P* <0.001) (Table 2). General community was 46.88% of participant posts and comments in the intervention and 62.86% of participant posts and comments in the control arm (*P* <0.001) (Table 2).

For the intervention arm, 33 participants were engaged (defined as reacted, commented, or posted) in week one, 29 in week two, 11 in week three, and 21 in week four. For the control arm, 30 participants were engaged in week one, 15 in week two, 16 in week three, and 7 in week four.

## DISCUSSION

As demonstrated by the decreased amount of misinformation in the intervention vs control group, results suggest that HOPE has the potential to reduce misinformation in social media groups with peer leaders. While this study looked to address COVID-19 vaccine misinformation, HOPE could be adapted to address misinformation for other public health issues. This has immediate public health implications as it can be used to both combat misinformation and disseminate information during public health crises.

## LIMITATIONS

Limitations include small sample size and short study duration. Our previous studies that used this intervention generally operated for 12 weeks. Neither the intervention nor control group participants posted much about facts, and what was posted was generally misinterpreted. This may be due to the peer leaders being the ones generally posting factual information. The short duration may also have been a factor in what participants could learn during that time. Future studies might explore ways to increase conversations about factual information.

There were also more posts and comments made by participants in the control group. This may be due to one outlier in group 4, who posted heavily (approximately 170 posts and 80 comments, which is more than the total of groups 1–3 combined). While this participant was later one of the ones removed from analysis, other people’s comments on their posts remained in the analysis. It is difficult to know whether the reduced misinformation in the intervention groups may have been due to them not wanting to post as much in groups with peer leaders. Past HOPE studies have found the intervention arm to generally have more posts and engagement compared to the control group,^11^,^15^ making it of interest to explore reasons for the control group having more in this study. Recruitment also targeted people who use Facebook and were employed as a frontline essential worker. This demographic may not necessarily represent the general population.

## CONCLUSION

Overall, results suggest that peer-led social media groups can be a powerful tool to help combat misinformation online and aid in addressing public health needs. Peer leaders can help shape the social norms within the group, reduce the spread of misinformation, and create a supportive community environment.

## Figures and Tables

**Table 1:** Example quotes of each topic. Each topic category is non-exclusive; so, posts and comments can potentially be labeled as all topics. Example quotes were shortened to the relevant text that represented a topic.

Topic	Example quote
General community	Hello! My name is [] and I'm a CNA in Kentucky.
Social support	We would be so hosed without CNAs, ya’ll rock!
COVID-19	Now there is talk about a new strain of covid called MU?
COVID-19: fact	Their are several and possibly more to come…the vaccines are waning and/or the new variants can evade the vaccine. Booster shots are planned to start in next few weeks https://www.who.int/.../act.../tracking-SARS-CoV-2-variants/
COVID-19: misinformation	When I start back in the ICU I will be taking ivermectin weekly prophylactically
COVID-19: experience	Looks like my employer is requiring the vaccine by oct 31st now. But I have antibodies still, I’ve had them since March when I had Covid.
COVID-19: opinion	My body my choice as to what I put in it and when. Period. That is one thing I will never change my mind on.
COVID-19: question	Has anyone been mandated by their employer yet?
Other: misinformation	I believe in the power of herbal remedies too.
Facts misinterpreted	That study shows a great several folds reduction of both infection and symptomatic disease in people with natural immunity. http://www.medrxiv.org/.../2021.08.24.21262415v1.full-text [link goes to a non-peer reviewed study]

*COVID-19*, coronavirus disease 2019; *CNA*, certified nursing assistant; *ICU*, intensive care unit.

**Table 2:** Coded conversation topics of participant posts and comments.

Group	1 (%)	2 (%)	Total Intervention (%)	3 (%)	4 (%)	Total Control (%)	Total Intervention vs Total Control P-value
Participant posts + comments (n)	67	93	160	61	254	315	<0.001
Number of reactions	60	137	197	43	142	185	
General community	22 (32.84%)	53 (56.99%)	75 (46.88%)	21 (34.43%)	177 (69.69%)	198 (62.86%)	<0.001
Social support	6 (8.96%)	13 (13.98%)	19 (11.88%)	2 (3.28%)	4 (1.57%)	6 (1.90%)	<0.001
COVID-19	43 (64.18%)	38 (40.86%)	81 (50.63%)	45 (73.77%)	126 (49.61%)	171 (54.29%)	0.45
COVID-19: fact	0 (0.00%)	3 (3.23%)	3 (1.88%)	5 (8.20%)	5 (1.97%)	10 (3.17%)	0.67
COVID-19: misinformation	8 (11.49%)	3 (3.23%)	11 (6.88%)	20 (32.79%)	40 (15.75%)	60 (19.05%)	<0.001
COVID-19: experience	25 (37.31%)	15 (16.13%)	40 (25.00%)	11 (18.03%)	46 (18.11%)	57 (18.10%)	0.08
COVID-19: opinion	21 (31.34%)	23 (24.73%)	44 (27.50%)	15 (24.59%)	73 (28.74%)	88 (27.94%)	0.92
COVID-19: question	3 (4.48%)	6 (6.45%)	9 (5.63%)	3 (4.92%)	6 (2.36%)	9 (2.86%)	0.14
Other: misinformation	3 (4.48%)	2 (2.15%)	5 (3.13%)	0 (0.00%)	7 (2.76%)	7 (2.22%)	0.55
Facts misinterpreted	0 (0.00%)	3 (3.23%)	3 (1.88%)	4 (6.56%)	4 (1.57%)	8 (2.54%)	0.65

*COVID-19*, coronavirus disease 2019.
